# Relevance of Interleukins 6 and 8 Single Nucleotide Polymorphisms in Prostate Cancer: A Multicenter Study

**DOI:** 10.1155/2021/3825525

**Published:** 2021-07-06

**Authors:** Amany A. Ghazy, Mohammed Jayed Alenzi

**Affiliations:** ^1^Department of Pathology (Microbiology and Immunology Unit), College of Medicine, Jouf University, Sakaka, Saudi Arabia; ^2^Departments of Microbiology and Medical Immunology, Faculty of Medicine, Kafr El-Sheikh University, Kafr El-Sheikh, Egypt; ^3^Department of Surgery, College of Medicine, Jouf University, Sakaka, Saudi Arabia

## Abstract

The diverse roles of cytokines as IL-6 and IL-8 have been studied in terms of their SNPs in many diseases but their role in prostate cancer (PCa) is still uncertain. *Aim*. To determine the relevance of IL-6 rs1800795 SNP and/or IL-8 rs2227306 SNP with prostate cancer's risk. *Subjects and Methods*. 40 PCa patients, 40 benign prostate hyperplasia (BPH) patients, and 40-age-matched-control group were enrolled in the study. Genotyping of IL-6 rs1800795 (G/C) SNP and IL-8 rs2227306 (C/T) SNP was determined using real-time PCR. *Results*. High frequency of IL-6 rs1800795GG and IL-8 rs2227306CC genotypes was noticed among PCa patients with associated OR 10.091 and 8.143, respectively. Comparisons based on allele frequencies revealed that IL-6G and IL-8C alleles are more frequent among PCa patients than other groups. Presence of IL-6 rs1800795G and IL-8 rs2227306C alleles in the same patient increase PCa risk by 16.7 times. Statistical correlations between PSA ratio and both of IL-6 and IL-8 SNP did not show any significant relation among PCa patients. *Conclusion*. IL-6 rs1800795G and IL-8 rs2227306C alleles could be considered risk factors for PCa development, particularly if presented together. However, no relation was found between both cytokines SNP and severity of prostate cancer.

## 1. Background

Benign prostate hyperplasia (BPH) and prostate cancer (PCa) are chronic, age-related, heterogeneous disorders with a wide variety of clinical presentations and high prevalence among men [1–4]. PCa results in high morbidity and mortality due to rapid progression to metastasis and the emergence of therapeutic resistance [5, 6].

According to Globocan, prostate cancer is considered one of the top ten cancers in Saudi Arabia and Egypt with an age-standardized incidence rate (ASR) of 7/100 000 and 13.9/100 000, respectively [2].

Statistics from American Association for Cancer Research (AACR) showed that PCa incidence is increasing annually. This incidence varies significantly between different ethnic groups and regions all over the world [7]. Thus, it is important to identify specific biomarkers associated with the progression of PCa [4]. Several studies have been conducted to investigate the molecular mechanism underlying PCa pathogenesis; some reported that there are specific genes that contribute to PCa risk [2]; others have implicated inflammation as a driver of PCa development [6]; and others stated that genetic mutation and single nucleotide polymorphism (SNP) have a significant role in the progression and severity of PCa but their results were dissimilar in the different populations [2, 8–11]^.^

Some cytokines were found to be involved in the etiopathogenesis of PCa by inducing growth, proliferation, and metastasis that occur during the course of the disease. Interleukin-6 (IL-6) is a pleiotropic cytokine that has multiple roles as induction and maintenance of inflammation, stimulation of adaptive immunity, and some autoimmune processes [11, 12]. IL-8 is known to have a role in immune surveillance, recruitment, and degranulation of neutrophils, inflammation, and angiogenesis [13]. It is overexpressed in many types of cancer as it has tumorigenic, and proangiogenic properties [5]. Recently, the diverse roles of IL-6 and IL-8 have been studied in terms of their SNPs in many diseases but their role in PCa is still uncertain.

Thus, the current study aimed to determine the possible association of IL-6 rs1800795 SNP, and/or IL-8 rs2227306 SNP with prostate cancer's risk and/or severity.

## 2. Subjects and Methods

### 2.1. Study Design

The current research is a case-control study enrolling 120 participants divided into 3 groups: group 1, 40 patients with pathologically confirmed PCa; group 2, 40 patients with BPH; and group 3, 40 age-matched healthy controls. Participants were attending Urology Department, Prince Mutaib bin Abdulaziz Hospital, Sakaka, and Kafr El-Sheikh University Hospital, Kafr El-Sheikh. Patients with autoimmune disease, renal decompensating, and/or secondary PCa were excluded from the study.

### 2.2. Ethical Considerations

Ethical approval (no. 06–03/41, 2019) was obtained from the Local Committee of Bioethics of Jouf University. The study adopts the ethical guidelines of the 1975 Declaration of Helsinki.

Informed written consent was collected from all participants with guarantees of confidentiality. There was no risk to the participants. Results of biochemical investigation and ultrasound were taken from patients' files. The blood samples used in DNA extraction and PCR were taken from the leftover samples in EDTA tubes.

### 2.3. Laboratory Investigations

Serum levels of urea, creatinine, total prostate-specific antigen (PSA), and PSA ratio were measured for all participants.

### 2.4. Genotyping of IL-6 rs1800795 (G/C) SNP and IL-8 rs2227306 (C/T) SNP

Blood samples in EDTA tubes were used for DNA extraction by spin columns of QIAamp DNA Blood Mini Kit (Applied Biosystems-Life Technologies), according to the manufacturer instructions [2, 14]. DNA concentration and purity were determined using nanodrop before real-time PCR. IL-6 rs1800795 and IL-8 rs2227306 genotypes were determined using step one real-time PCR (Applied Biosystem-Life Technologies, Carlsbad, California, USA). For each SNP, 2 *μ*l genomic DNA, 1.25 *μ*l TaqMan SNP genotyping assay (IL-6 rs1800795 SNP kit or IL-8 rs2227306 SNP kit), 12.5 *μ*l TaqMan PCR master mix, and 9.25 *μ*l DNase-free water were mixed in PCR tubes. The reaction volume was 25 *μ*l and the thermal cycler conditions were adjusted as follows: 95°C for 10 minutes for initial denaturation, followed by 40 cycles of amplification (92°C for 15 sec., 60°C for 1 min., and 72°C for 30 sec.), then a final extension at 72°C for 7 min. Results analysis depends on the fluorescence signals emitted from each sample; FAM dye points to homozygosity for the wild alleles (IL-6 rs1800795 CC, IL-8 rs2227306TT), VIC dye specifies homozygosity for the mutant alleles (IL-6 rs1800795GG, IL-8 rs2227306CC), and if both increase, this indicates heterozygosity (IL-6 rs1800795GC, IL-8 rs2227306 CT) [2, 15].

### 2.5. Statistical Analysis of the Data

Data were analyzed using version 20.0. of IBM SPSS software package (Armonk, NY: IBM Corp). The normality of the distribution of variables was verified by Kolmogorov–Smirnov. Categorical variables of the studied groups were compared using the Chi-square test. Comparisons of normally and abnormally distributed quantitative variables were done using Student's *t*-test and Mann–Whitney *U* test, respectively. Odds ratio (OR) was used to calculate the ratio of the odds and 95% confidence interval of an event occurring in one risk group to that in the nonrisk group. The population of the studied sample was explored to find its equilibrium with the Hardy–Weinberg equation. The significance of the results was judged at the 5% level.

## 3. Results

### 3.1. Subjects Demographic Data

The mean age of PCa patients, BPH patients, and controls was 59.9 ± 9.2, 56 ± 10.3, and 50 ± 8, respectively. There was not any statistical difference between the studied groups (*p* = 0.1).

### 3.2. Biochemical Investigations

The mean serum levels of urea and creatinine among PCa patients were significantly increased among PCa patients in comparison to the control group (*p* < 0.001^*∗*^). PCa patients showed a significant increase in total PSA and PSA ratio (*p* < 0.001^*∗*^) (Table 1).

### 3.3. Genotyping of IL-6 and IL-8 SNPs

IL-6 and IL-8 genotypes frequencies were in agreement with the Hardy–Weinberg equation (HWE) in PCa patients and controls because there was an insignificant difference between the observed and expected frequencies (*p* > 0.05, Table 2).

Regarding the distribution of IL-6 rs1800795C/G genotypes, GG genotype was more frequent among PCa patients in comparison with BPH patients and control groups (45% vs. 15% and 7.5%, resp.). Patients carrying GG genotype are at higher risk to develop PCa than those carrying CC genotype (Table 3). Allelic discrimination of IL-8 rs2227306 (C/T) SNP showed a higher frequency of CC genotype among PCa patients in comparison with BPH patients and control groups (30% vs, 7.5% and 5%, resp., *p* = 0.010^*∗*^). Patients carrying CC genotype are 8 times at higher risk to develop PCa than those carrying TT genotype (Table 3).

Such difference was more obvious when the comparison was based on allele frequencies as IL-6 rs1800795G allele was more frequent among PCa patients in comparison with BPH patients and control groups (62.5% vs. 46.3% and 22.5%, resp.). Patients carrying IL-6 rs1800795G allele are at 5.7 times more risk to develop PCa than those carrying C allele (Table 4). In addition, IL-8 rs2227306C allele is more frequent among PCa patients when compared to BPH patients and control groups (51.3% vs. 37.5% and 25%, resp.). Patients carrying IL-8 rs2227306C are 3.154 times at higher risk to develop PCa than those carrying T allele (Table 4).

Determination of the frequency of IL-6 and IL-8 SNPs haplotypes showed that the presence of IL-6 rs1800795G and IL-8 rs2227306C alleles in the same patient increase PCa risk by 17.7 times *p* < 0.001^*∗*^) (Table 5).

### 3.4. PSA Levels and the Haplotypes of IL-6 rs1800795 and IL-8 rs2227306 SNPs

Statistical correlations between PSA ratio, IL-6rs1800795 SNP, IL-8 rs2227306 SNP and haplotypes of both SNPs among PCa and BPH patients did not show any significant relation (Table 6).

## 4. Discussion

Prostate cancer is one of the most common tumors among men, with a high mortality rate. Growing evidence incriminates inflammation as a driver of PCa initiation and progression. Environmental exposure to infections, mutagenic agents, and genetic variations predispose to inflammation and increase the expression of inflammatory cytokines as IL-6 and TGF-*β* [6].

IL-6 and IL-8 were reported to be incriminated in the initiation and progression of prostate tumorigenesis, as IL-6 induces C-reactive protein (CRP) production [12]; promotes PCa cell survival, proliferation, invasion; enhances the progression of PCa to the castration-resistant state; and inhibits apoptosis [16, 17]. At the same time, IL-8 was found to enhance androgen independence, tumor growth, angiogenesis, and chemoresistance [13].

Recently, the diverse roles of IL-6 and IL-8 have been studied in terms of their SNPs. Different studies have examined the effect of IL-6 SNP and/or IL-8 SNP on diseases' risk, activity, and progression. Results showed marked ethnicity-associated disparities and diverse findings [12, 16–23]. For instance, a meta-analysis of European and Asian studies on IL-6 SNP and rheumatoid arthritis (RA) risk showed that IL-6 SNP was significantly associated with RA in Asian patients but not in the Caucasian population [20]. Another study has reported a strong association between IL-6 SNP and hand joint damage among Mexican patients [19]. In addition, vitamin D receptor (VDR) rs7975232 SNP was reported to predict PCa risk in African American men [23]. However, Cao et al. [24] have reported that PCa antigen 3 SNP (rs544190G > A) was associated with metastatic prostate cancer among the European descent but has no association in the Eastern Chinese population. These results exposed the possible ethnicity-associated polymorphisms.

Accordingly, the current research intended to appraise the relevance of IL-6 rs1800795 SNP and IL-8 rs2227306 (C/T) SNP with PCa, with some emphases on disease risk and severity. Odds ratio (OR) with its 95% confidence interval (CI) was calculated to estimate the association's strength. Results showed that the three genotypes (CC, CG, and GG) of IL-6 rs1800795 SNP were presented in all studied groups with different frequencies where GG genotype was more frequent among PCa patients in comparison with BPH patients and control groups. Patients carrying IL-6 rs1800795GG genotype were at higher risk to develop PCa than those carrying CC genotype [OR1 (95%CI) = 10.091 (2.665–38.204), *p* = 0.001^*∗*^]. Such difference was more obvious when comparing allele frequencies where IL-6 rs1800795G allele showed a higher frequency in PCa patients (susceptibility allele) and the associated OR was 5.741 (*p* < 0.001^*∗∗*^). This association can be explained by the fact that such IL-6 promotor SNP may influence IL-6 gene expression and serum level [13]. This in turn will promote inflammatory pathways in PCa patients [6].

These results were in agreement with a study on the European American men where the C allele of IL-6 rs1800795 (-174, G/C) SNP, a known functional variant, was associated with increased PCa risk [25]. Moreover, a study on a northwest Iranian population has reported that IL-6 rs1800795 (-174, G/C) SNP represents a good predictor for PCa susceptibility and bone metastasis as it was concomitant with increased IL-6 production and PCa risk. They have explained this by the fact that the promoter SNPs -174G > C (rs1800795) can modify the transcriptional pattern of IL-6 gene affecting its production and in turn inflammatory processes that can predispose to PCa development [11].

Recently, a meta-analysis was performed on 97 original research covering IL-6 promoter SNPs and noticed a significant association with cancer risk and prognosis. Subgroup analysis showed that IL-6 rs1800795 SNP was significantly related to an increased possibility of cancer of breast, cervix, prostate, colon, and/or lung but not gastric cancer or multiple myeloma [26].

On the other hand, some studies could not find any link between IL-6 rs1800795 (-174, G/C) SNP and PCa risk [18, 27]. A study in Han people (Hubei region) has reported that PCa development and progression was not linked to rs1800795 (-174, G/C) SNP of IL-6 promoter region [27]. In a study in Eastern Croatia, no association was noticed between IL-6 rs1800795 (-174, G/C) SNP and PCa risk. They suggested that the distribution of IL-6-174 SNP may vary between the different ethnic groups [18].

Regarding IL-8, it was reported that IL-8 levels are significantly upregulated in PCa tissues. It is suggested to exhibit direct oncogenicity via induction of tumor cells proliferation, invasion, and attenuation of apoptosis [5]. IL-8 secretion from prostate epithelial cells is strongly related to the aggressiveness of the tumor [6]. Thus, analysis of IL-8 gene polymorphisms could be valuable in affecting its expression level.

In the current study, allelic discrimination of IL-8 rs2227306 (C/T) SNP revealed a higher frequency of CC genotype among PCa patients in comparison with the other groups and the associated OR was 8.143 (*P* = 0.009^*∗*^). This difference was more obvious when comparing allele frequencies. IL-8 rs2227306C allele showed higher frequency in PCa patients compared to BPH patients and control groups (51.3% vs. 37.5% and 25%, resp.). These findings suggest that IL-8 rs2227306T allele could be a protective allele [OR (95%CI) = 0.317 (0.162–0.619), *p* = 0.001^*∗*^], while IL-8 rs2227306C allele might be a risk allele [OR (95%CI) = 3.154 (1.615–6.161), *p* = 0.001^*∗*^]. Statistical analysis of associated IL-6 and IL-8 SNPs haplotypes frequency showed that the presence of IL-6 rs1800795G and IL-8 rs2227306C alleles in the same patient increase PCa risk by 16.7 times (*p* < 0.001^*∗*^). However, correlation analysis between PSA ratio and both of IL-6 and IL-8 SNP did not show any significant relation among PCa patients. This indicates that these genotypes or haplotypes did not have any effect on the severity of PCa.

In partial agreement with our results, a meta-analysis has explored the roles of IL-8 rs2227306 SNP and cancer risk in 22 case-control studies; they identified a significant decrease of hepatocellular carcinoma risk with IL-8 rs2227306T/C polymorphism (OR = 0.72, 95% CI = 0.56–0.91, *p* = 0.007^*∗*^) [28]. This indicates the protective effect of T allele. Others have evaluated the association between IL-8 rs4073 SNP and PCa risk and could not find any association [29, 30]. Franz et al. [30] have reported that IL-8 SNP could influence its production. They have investigated the influence of the IL-8-251T/A SNP on PCa susceptibility and clinicopathological characteristics in Brazilian patients but did not find any association. These divergences in results could be related to the heterogeneity of PCa [24], location of the SNP on the gene [31], and/or ethnic differences [24].

Finally, the main limitations in our study are the inability to screen larger panels of alleles and we could not relate our results with the family cancer history of patients. Thus, further large-scale studies concerning genomic sequencing, gene-environment, and/or gene-gene interaction are recommended, particularly in families with a history of PCa.

## 5. Conclusion

IL-6 rs1800795G and IL-8 rs2227306C alleles may be considered risk factors for PCa, particularly if presented together. However, no relation was found between both cytokines SNP and the severity of PCa.

## Figures and Tables

**Table 1:** Comparison of PSA levels of the affected and the control groups.

Prostate-specific antigen (PSA)	PCa patients (*n* = 40)	BPH patients (*n* = 40)	Control (*n* = 40)	*p*
S. urea (mg/dl)	26.6 ± 5.7	14.3 ± 2.8	12.7 ± 3.1	<0.001^*∗*^
S. creatinine (mg/dl)	1.2 ± 0.2	0.9 ± 0.1	0.8 ± 0.3	<0.001^*∗*^
Total PSA (ng/ml)	4.8 ± 0.9	2.5 ± 0.9	1.8 ± 0.5	<0.001^*∗*^
PSA ratio %	37 ± 7.5	17.5 ± 3.4	10.5 ± 3.1	<0.001^*∗*^

Data are represented by mean ± SD, *p*: *p* value for comparing between the studied groups. ^*∗*^Statistically significant at *p* ≤ 0.05.

**Table 2:** Distribution of genotypes of IL-6 rs1800795 and IL-8 rs2227306 SNPs in the affected and the control groups.

	Cancer (*n* = 40)		BPH (*n* = 40)		Control (*n* = 40)	
	Observed	Expected	Observed	Expected	Observed	Expected
IL-6 rs1800795						
CC	8	5.6	9	11.6	25	24.0
CG	14	18.8	25	19.9	12	14.0
GG	18	15.6	6	8.6	3	2.0
*χ* ^2^ (p)	2.567 (0.109)		2.643 (0.104)		0.782 (0.377)	

IL-8 rs2227306						
CC	12	10.5	3	5.6	2	2.5
CT	17	20.0	24	18.8	16	15.0
TT	11	9.5	13	15.6	22	22.5
*χ* ^2^ (p)	0.894 (0.344)		3.136 (0.077)		0.178 (0.673)	

**Table 3:** Comparison of genotype ratios of IL-6 rs1800795 and IL-8 rs2227306 SNPs in the affected and the control groups.

	Frequencies of genotypes			Cancer versus control®		BPH versus control®	
	Cancer (*n* = 40)	BPH (*n* = 40)	Control (*n* = 40)	*p*_1_	OR_1_ (95%CI) (LL–UL)	*p*_2_	OR_2_ (95%CI) (LL–UL)
IL-6 rs1800795							
CC versus (CG + GG)	8 (20%)	9 (22.5%)	25 (62.5%)	<0.001^*∗*^	0.1500 (0.055–0.410)	<0.001^*∗*^	0.174 (0.065–0.464)
CG versus (CC + GG)	14 (35%)	25 (62.5%)	12 (30%)	0.633	1.256 (0.492–3.209)	0.004^*∗*^	3.889 (1.533–9.868)
GG versus (CC + CG)	18 (45%)	6 (15%)	3 (7.5%)	0.001^*∗*^	10.091 (2.665–38.204)	0.297	2.177 (0.504–9.391)

IL-8 rs2227306							
CC versus (CT + TT)	12 (30%)	3 (7.5%)	2 (5%)	0.009^*∗*^	8.143 (1.686–39.319)	0.646	1.541 (0.243–9.755)
CT versus (CC + TT)	17 (42.5%)	24 (60%)	16 (40%)	0.820	1.109 (0.455–2.701)	0.076	2.250 (0.920–5.504)
TT versus (CC + CT)	11 (27.5%)	13 (32.5%)	22 (55%)	0.014^*∗*^	0.310 (0.122–0.789)	0.045^*∗*^	0.394 (0.159–0.978)

®: reference group, OR: odds ratio, CI: confidence interval, UL: upper limit, LL: lower limit. OR_1_, *p*_1_: odds ratio and *p* value for comparing between cancer and control. OR_2_, *p*_2_: odds ratio and *p* value for comparing between BPH and control. ^*∗*^Statistically significant at *p* ≤ 0.05.

**Table 4:** Comparison of allele frequencies of IL-6 rs1800795 and IL-8 rs2227306 SNPs in the three studied groups.

	Allele frequency			Cancer versus control®		BPH versus control®	
	Cancer (*n* = 80)	BPH (*n* = 80)	Control (*n* = 80)	*p*_1_	OR_1_ (95%CI) (LL–UL)	*p*_2_	OR_2_ (95%CI) (LL–UL)
IL-6 rs1800795							
C versus G	30 (37.5%)	43 (53.8%)	62 (77.5%)	<0.001^*∗*^	0.174 (0.087–0.348)	0.002^*∗*^	0.337 (0.170–0.669)
G versus C	50 (62.5%)	37 (46.3%)	18 (22.5%)	<0.001^*∗*^	5.741 (2.871–11.480)	0.002^*∗*^	2.964 (1.495–5.877)

IL-8 rs2227306							
C versus T	41 (51.3%)	30 (37.5%)	20 (25%)	0.001^*∗*^	3.154 (1.615–6.161)	0.090	1.800 (0.913–3.549)
T versus C	39 (48.8%)	50 (62.5%)	60 (75%)	0.001^*∗*^	0.317 (0.162–0.619)	0.090	0.556 (0.282–1.095)

®: reference group, OR: odds ratio, CI: confidence interval, UL: upper limit, LL: lower limit. OR_1_, *p*_1_: odds ratio and *p* value for comparing between cancer and control. OR_2_, *p*_2_: odds ratio and *p* value for comparing between BPH and control. ^*∗*^Statistically significant at *p* ≤ 0.05.

**Table 5:** Comparison of haplotype frequencies of IL-6 rs1800795 and IL-8 rs2227306 SNPs in the affected and the control groups.

Haplotype IL-6 rs1800795 and IL-8 rs2227306	Haplotype frequencies (%)			Cancer versus control®		BPH versus control®	
	Cancer (*n* = 80)	BPH (*n* = 80)	Control (*n* = 80)	*p*_1_	OR_1_ (95%CI) (LL–UL)	*p*_2_	OR_2_ (95%CI) (LL–UL)
CC versus (CT + GC + GT)	17 (21.3%)	26 (32.5%)	18 (22.5%)	0.848	0.930 (0.439–1.967)	0.158	1.658 (0.821–3.350)
CT versus (CC + GC + GT)	13 (16.3%)	17 (21.3%)	44 (55%)	<0.001^*∗*^	0.159 (0.076–0.333)	<0.001^*∗*^	0.221 (0.110–0.442)
GC versus (CC + CT + GT)	24 (30%)	4 (5%)	2 (2.5%)	<0.001^*∗*^	16.714 (3.794–73.631)	0.414	2.053 (0.365–11.538)
GT versus (CC + CT + GC)	26 (32.5%)	33 (41.3%)	16 (20%)	0.075	1.926 (0.937–3.958)	0.004^*∗*^	2.809 (1.387–5.689)

®: reference group, OR: odds ratio, CI: confidence interval, UL: upper limit, LL: lower limit. OR_1_, *p*_1_: odds ratio and *p* value for comparing between cancer and control. OR_2_, *p*_2_: odds ratio and *p* value for comparing between BPH and control. ^*∗*^Statistically significant at *p* ≤ 0.05.

**Table 6:** Relation between PSA ratio with IL-6 rs1800795 and IL-8 rs2227306 in each patient group.

		*N*	Min.–Max.	PSA ratio % Mean ± SD	Median	*H*	*p*
Cancer group	IL-6 rs1800795						
	CC	**8**	28.8–57.5	36.5 ± 8.8	35	2.089	0.352
	CG	**14**	27.2–46.9	35.1 ± 6.9	31.7		
	GG	**18**	29.1–56.6	38.7 ± 7.3	37.6		
	IL-8 rs2227306						
	CC	**12**	27.3–47.4	35.5 ± 7.3	33.6	1.262	0.532
	CT	**17**	29.1–57.5	37.6 ± 8.4	36		
	TT	**11**	27.2–46.9	37.6 ± 6.3	40		
	Haplotypes of IL-6 rs1800795 and IL-8 rs2227306						
	CC	**17**	27.3–57.5	35.1 ± 7.7	32.5	2.873	0.412
	CT	**13**	27.2–57.5	36.8 ± 8	35		
	GC	**24**	27.3–56.6	37.2 ± 7.8	35.5		
	GT	**26**	27.2–56.6	38.1 ± 6.8	40		

BPH group	IL-6 rs1800795						
	CC	**9**	15.8–24	18.2 ± 2.7	17.2	0.578	0.749
	CG	**25**	10.5–22.4	17.6 ± 3.5	17.2		
	GG	**6**	10.5–21	16.4 ± 4.2	17.7		
	IL-8 rs2227306						
	CC	**3**	10.5–16.5	14.3 ± 3.3	16	2.726	0.256
	CT	**24**	10.5–24	17.7 ± 3.5	17.2		
	TT	**13**	12.1–22.4	18 ± 3.2	19.2		
	Haplotypes of IL-6 rs1800795 and IL-8 rs2227306						
	CC	**26**	10.5–24	17.6 ± 3.4	17.2	4.502	0.212
	CT	**17**	12.5–24	18.3 ± 2.9	19.2		
	GC	**4**	10.5–16.5	13.5 ± 3.5	13.5		
	GT	**33**	10.5–22.4	17.6 ± 3.5	18.8		

*H*: Kruskal–Wallis test, *p*: *p* value for association between different categories.

## Data Availability

All the data are available from the corresponding author upon request.

## Additional Points

Research highlights are as follows: (1) IL-6 rs1800795 G and IL-8 rs2227306 C alleles could be considered risk factors for PCa development, particularly if presented together. (2) Patients carrying IL-6 rs1800795G allele are 5.741 times at higher risk to develop PCa than those carrying C allele. (3) Patients carrying IL-8 rs2227306 C are 3.154 times at higher risk to develop PCa than those carrying T allele. (4) Association of IL-6 rs1800795G and IL-8 rs2227306C alleles in the same patient increase PCa risk by 16.7 times. (5) Correlation analysis between PSA ratio, and both of IL-6 and IL-8 SNP did not show any significant relation among PCa patients.
